# The impact of patient involvement on participant opinions of information sheets

**DOI:** 10.1192/bjo.2022.627

**Published:** 2023-01-09

**Authors:** Georgie Hudson, Sonja M. Jansli, Daniel Morris, Til Wykes, Sagar Jilka

**Affiliations:** Institute of Psychiatry, Psychology & Neuroscience, King's College London, London, UK; South London and Maudsley NHS Foundation Trust, London, UK; and Division of Psychology and Language Sciences, University College London, London, UK; Institute of Psychiatry, Psychology & Neuroscience, King's College London, London, UK; and South London and Maudsley NHS Foundation Trust, London, UK; Institute of Psychiatry, Psychology & Neuroscience, King's College London, London, UK; Institute of Psychiatry, Psychology & Neuroscience, King's College London, London, UK; South London and Maudsley NHS Foundation Trust, London, UK; and Warwick Medical School, University of Warwick, Coventry, UK

**Keywords:** Consent and capacity, patient and public involvement, accessibility, information sheets, readability

## Abstract

**Background:**

Patient and public involvement (PPI) groups can provide valuable input to create more accessible study documents with less jargon. However, we don't know whether this procedure improves accessibility for potential participants.

**Aims:**

We assessed whether participant information sheets were rated as more accessible after PPI review and which aspects of information sheets and study design were important to mental health patients compared with a control group with no mental health service use.

**Method:**

This was a double-blind quasi-experimental study using a mixed-methods explanatory design. Patients and control participants quantitatively rated pre- and post-review documents. Semi-structured interviews were thematically analysed to gain qualitative feedback on opinions of information sheets and studies. Two-way multivariate analysis of variance was used to detect differences in ratings between pre- and post-review documents.

**Results:**

We found no significant (*P* < 0.05) improvements in patient (*n* = 15) or control group (*n* = 21) ratings after PPI review. Patients and controls both rated PPI as of low importance in studies and considered the study rationale as most important. However, PPI was often misunderstood, with participants believing that it meant lay patients would take over the design and administration of the study. Qualitative findings highlight the importance of clear, friendly and visually appealing information sheets.

**Conclusions:**

Researchers should be aware of what participants want to know about so they can create information sheets addressing these priorities, for example, explaining why the research is necessary. PPI is poorly understood by the wider population and efforts must be made to increase diversity in participation.

Participant information sheets need to be clear and jargon-free to achieve true informed consent.^1^ This can be facilitated by non-tokenistic^2^ patient and public involvement (PPI) when creating them.^3^ The Feasibility and Support to Timely Recruitment for Research (FAST-R; https://www.maudsleybrc.nihr.ac.uk/patients-public/support-for-researchers/) service offers access to trained mental health patients and carers to improve participant-facing documents such as participant information sheets. This service was set up by the Mental Health Research Network in London in 2011 and is now organised and funded by the National Institute of Health and Care Research (NIHR) Maudsley Biomedical Research Centre. The PPI review highlights potential stumbling blocks to ethical approval, for example, by ensuring clear and accessible language in participant-facing documents and including information needed for genuinely informed consent.^4^ The PPI group reviews documents and returns them within 7 working days. In a recent study, we found that reviewed documents contained less jargon and were preferred over the original versions in a blind test.^3^ PPI reviewers highlighted issues relating to document clarity, data protection, vocabulary, study design and presentation, and also raised issues that are overlooked by regulatory bodies. The US Food and Drug Administration recommend a readability grade of eight or lower (i.e. readable by someone aged 13 years.^5^) However mental health study information rarely meets this recommended reading grade.^1,3^ It is therefore vital to understand how this lowered accessibility will affect potential research participants, including those who use mental health services and those who do not. This study builds upon our previous work to understand the impact of PPI by investigating diverse research-naïve participants. Many such investigations have included participants who are not sufficiently ethnically diverse,^6^ so we specifically recruited an ethnically diverse group. We also investigated whether a lack of readability in information sheets was an issue for all potential participants or whether it was unique to mental health patients by comparing those with a mental health service history and people without (control participants).

## Method

### Design

This was a double-blind quasi-experimental study using a mixed-methods explanatory design investigating patients’ and controls’ opinions of participant information sheets from before and after PPI review. All participants were randomly assigned a group of three different participant information sheets to review from either before or after PPI review.

The authors assert that all procedures contributing to this work comply with the ethical standards of the relevant national and institutional committees on human experimentation and with the Helsinki Declaration of 1975, as revised in 2008. All procedures involving human subjects/patients were approved by the London-Brent Research Ethics Committee on 2 January 2020 (ref: 19/LO/1857).

### Participants

Two groups of participants were recruited:

- Research-naïve patients who had never been part of a PPI group were recruited via the South London and Maudsley NHS Foundation Trust Consent for Contact (C4C) register.^7^ C4C is a clinical database; people who are seen clinically are offered an opportunity to take part in research by registering with this database, and researchers are able to invite them to participate in research. Only patients who had never taken part in a research study were recruited.
- Control participants with no experience of using mental health services were recruited from the general public and through a university-wide newsletter, using purposive sampling to ensure diversity in age, ethnicity and gender.

Apart from a personal history of mental health service use, the inclusion criteria were the same for both groups: aged at least 18, fluent in English, ability to give informed consent, no experience of PPI consulting and access to the internet (owing to COVID-19 research regulations).

We aimed to recruit until we reached data saturation for the qualitative themes. We ensured that at least 50% of our sample were from ethnic minority backgrounds.

### Information sheets

Information sheets were a subsample of those reviewed by the PPI group and studied previously.^3^ Twenty-four participant information sheets from 12 mental health studies were included (12 before and 12 after PPI review) that were representative in terms of readability, complexity of the study topic, and number of changes suggested by the reviewers and implemented by the researchers after PPI review.^3^ The number of changes requested by PPI reviewers ranged from three to 28, with the percentage of these changes implemented ranging from 44% (4/9) to 100% (13/13). We ensured that the participant information sheets assigned to the two participant groups had an even distribution of all these variables.

### Measures

#### Demographic measures

Age, ethnicity, first language, gender, highest qualification, current employment status, and mental health diagnoses and duration (for patient participants) were collected.

#### Information sheet scoring

Each information sheet was rated on 11 Likert scale items (five points from ‘not at all’ to ‘very’; e.g. ‘Is the language in the document easy to understand?’). Some items were reverse-scored and recoded during data analysis so that higher scores always represented better ratings. This measure was based on the qualitative findings of Staley et al^8^ that PPI review comments fall into seven categories. As two of these categories were exclusive to protocols (recruitment and research design), our 11 questions mapped onto the remaining five categories:

- quality of written information (five questions);
- informed consent process (one question);
- care and protection of participants (three questions);
- data protection and confidentiality (one question);
- practical aspects of the study (one question).

A composite score was created, consisting of the total individual scores for each scoring sheet.

#### Study feature importance questionnaire

Participants rated, on a five-point Likert scale (one: ‘not important’; five: ‘very important’), how important they thought it was for an information sheet to describe the following study features: (a) detailed methods, (b) PPI, (c) data protection and confidentiality, (d) providing background to the research, (e) clear explanations of why the research is necessary, (f) the research filling a gap in the literature and (g) large sample sizes. Participants then ranked these study features with respect to importance from one to seven, with one being the most important and seven the least. These features were based on those found to be most important to PPI reviewers in previous work.^3,8^

### Procedure

The information sheets included in the study were all assigned a unique identifier which did not reveal their status as either *before* or *after* review. One author randomly grouped the information sheets into sets of three, with at least one information sheet in each set being: (a) an original information sheet submitted for PPI review by the researchers, and (b) a revised information sheet after PPI review. No set contained the same study information sheet from both before and after review. After written informed consent was obtained, each participant was randomly assigned to, and emailed, one set of three participant information sheets. This randomisation was double-blind so neither the researcher communicating with the participants nor the participants were aware of whether each information sheet had been reviewed by FAST-R. The allocation of information sheets to participants was conducted by one author who had no contact with participants. In order to minimise bias and to prevent participants guessing which information sheet had been through PPI, participants were not informed that the information sheets they scored were of different versions. Participants completed the questionnaires in their own time and emailed them back to the research team. Each participant who returned completed questionnaires was then invited to take part in a semi-structured interview held virtually on Microsoft Teams and audio-recorded. During the interview, participants were asked about the reasons behind their scores and their opinions on PPI in the research process. At this point, participants were given a brief description of what PPI entailed. It was described how people with lived experience can act as an ‘advisory board’ and assist researchers with practical aspects of their study. It was clarified that PPI does not involve patients carrying out scientific research but guiding the research using their personal experiences. Participants were also asked their opinions on what constitutes a ‘good’ participant information sheet. See Supplementary material available at https://doi.org/10.1192/bjo.2022.627 for the interview topic guide.

### Quantitative analyses

#### Data robustness and context

We used independent *t*-tests to assess whether the information sheets were balanced across participant groups in terms of complexity (using reading grade^9^) and accessibility (using the number of jargon words and word count^3^).

Demographic differences between participant groups (patients versus control) were analysed using two-tailed analysis of variance (ANOVA) and chi-squared tests. Ethnicity was converted into a binary variable (White versus people of colour) owing to the small numbers, as per Jilka et al^10^ and O'Connor et al.^11^ Employment status was similarly converted (full-time employment versus any other employment status). Any differences were controlled in further analyses.

#### Differences between participant groups and review status

A two-tailed ANOVA was used to investigate whether the rating of study factor importance differed between participant groups. All 11 information sheet items and the composite scores were analysed using a two-way multivariate ANOVA to test for differences in scores depending on review status (before versus after PPI review) and between participant groups (patients versus controls). As two information sheets only had three changes after PPI review, we conducted a sensitivity analysis by removing these information sheets and re-running the multivariate ANOVA to investigate whether the findings were an artefact of a small number of changes.

All data analyses were carried out using SPSS version 27 for Windows.^12^

### Qualitative analysis

Interview transcripts were analysed thematically using the Braun and Clarke^13^ method also used in previous publications.^3,14^ Themes were inductively and independently extracted by two researchers, using the five-stage analysis framework of Pope et al:^15^ familiarisation with the raw data; identifying a thematic framework; applying the thematic framework; charting the data according to the thematic framework; and mapping and interpretation by defining concepts, mapping the range and nature of phenomena, and creating typologies. To minimise bias and maximise our inductive approach from an emic perspective,^16^ two patient researchers independently undertook inductive coding^17^ to categorise the codes into themes and subthemes. The two researchers created the final inductive framework together by discussing the similarities and differences between their frameworks and using the elements of the multiple coding approach.^18^ Theme names were decided collaboratively by the two researchers. Any discrepancies were resolved through discussion between the two researchers and independently checked by a third researcher. NVivo version 12 for Windows was used to manage the data.^19^

## Results

### Participant information

A total of 36 participants were recruited (*n* = 21 controls, *n* = 15 patients). Approximately 200 eligible patients were contacted, with a success rate of 7.5%. See Table 1 for the demographic breakdown. The groups did not differ on any demographic characteristic (see Supplementary material for a breakdown of the results). One patient withdrew halfway, resulting in missing data for one information sheet, study feature importance and demographic questionnaires.

**Table 1:** Breakdown of participant characteristics

Characteristic	All participants (*N* = 35)^a^	Patient group (*n* = 14)^a^	Control group (*n* = 21)
Gender, *n* (%)			
Female	23 (65.7%)	11 (78.6%)	12 (57.1%)
Male	12 (34.3%)	3 (21.4%)	9 (42.9%)
Age in years, mean (s.d.)	36.57 (14.30) Range: 19–72	39.43 (17.14) Range: 19–72	33.05 (11.07) Range: 21–59
Ethnicity, *n*(%)			
Asian/Asian British	9 (25.7%)	2 (14.3%)	7 (33.3%)
Black/African/Caribbean/Black British	9 (25.7%)	5 (35.7%)	4 (19.0%)
Mixed/multiple ethnic groups	3 (8.6%)	–	3 (14.3%)
White	14 (40.0%)	7 (50.0%)	7 (33.3%)
Native English speaker, *n* (%)	29 (82.9%)	13 (92.9%)	16 (76.2%)
Highest qualification, *n* (%)			
GCSE or equivalent	2 (5.7%)	2 (14.3%)	–
A-Level or equivalent	8 (22.9%)	3 (21.4%)	5 (23.8%)
Bachelor's degree	15 (42.9%)	6 (42.9%)	9 (42.9%)
Postgraduate qualification	10 (28.6%)	3 (21.4%)	7 (33.3%)
Employment status, *n* (%)			
Employed full-time	16 (45.7%)	4 (28.6%)	12 (57.1%)
Employed part-time	5 (14.3%)	1 (7.1%)	4 (19.0%)
Unemployed	3 (8.6%)	3 (21.4%)	–
Self-employed	3 (8.6%)	2 (14.3%)	1 (4.8%)
Full-time student	5 (14.3%)	2 (14.3%)	3 (14.3%)
Other	3 (8.6%)	2 (14.3%)	1 (4.8%)
Currently a student, *n* (%)	11 (31.4%)	3 (21.4%)	8 (38.1%)
Mental health diagnoses, *n* (%)			
Depression	10 (71.4%)	
Bipolar disorder	3 (21.4%)	
Anxiety disorder	2 (14.3%)	
Obsessive compulsive Disorder	2 (14.3%)	
Post-traumatic stress disorder	2 (14.3%)		N/A
Schizophrenia or psychosis	2 (14.3%)	
Eating disorder	1 (7.1%)	
Panic disorder	1 (7.1%)	
None of the above	1 (7.1%)	
Years with mental health diagnosis, mean (s.d.) (*N* = 13)	10.92 (10.35) Range: 1–30		N/A

a. One patient did not provide demographic information; percentages have been calculated accordingly.

### Quantitative results

#### Characteristics of information sheets between participant groups

We found no statistically significant differences in the reading grade (*t*(105) = 0.14, *P* = 0.890), number of jargon words (*t*(105) = 0.72, *P* = 0.677) or word count (*t*(105) = −0.12, *P* = 0.904) of information sheets reviewed between the participant groups, indicating equity in the distribution of information sheet characteristics across groups.

#### Differences in ratings of importance of study features

‘A clear explanation of why the research is necessary’ was rated as the most important study feature by both groups. There were no statistically significant differences in ratings between groups, and both groups ranked patient involvement low. Table 2 shows the participants’ importance rankings of various aspects of studies.

**Table 2:** Aspects of studies, ranked by importance (left to right), split by participant group type. Lower scores represent a rating of higher importance. Involving patients was ranked fifth and sixth by patients and controls

	A clear explanation of why the research is necessary	A clear explanation of how data will be collected for the study	Providing background to the research	Showing that the research fills a gap in current knowledge	Having a large sample size/number of people participating in the study	Involving patients in the design and/or running of the study	Explaining to participants how the study was designed
Overall average ranking	2.14	2.71	3.60	4.37	4.54	4.69	4.83
Overall ranking	1	2	3	4	5	6	7
Patient ranking	1	2	3	4	7	5	6
Control ranking	1 (tied)	1 (tied)	3	5	4	6	7

#### Do participants prefer post-PPI-review information sheets?

There was no main effect of information sheet review status on participant ratings (F(11,93) = 0.93, *P* = 0.516, Wilk's Λ = 0.90, partial η2 = 0.10). There were also no significant differences between pre- and post-review information sheet ratings on the individual items (Table 3).

**Table 3:** Mean score and standard deviation for the participant information sheets, split by participant group and whether the information sheet was before or after PPI review. Higher scores represent better ratings

Rating	Mean score (s.d.)					
	Overall		Patient group		Control group	
	Before	After	Before	After	Before	After
Ease of language to understand	4.61 (0.62)	4.50 (0.72)	4.54 (0.76)	4.83 (0.38)	4.67 (0.48)	4.30 (0.79)
Ease of study to understand	4.37 (0.74)	4.33 (0.88)	4.54 (0.76)	4.50 (0.79)	4.24 (0.71)	4.23 (0.94)
Structure	4.46 (0.82)	4.27 (0.89)	4.31 (1.01)	4.56 (0.62)	4.58 (0.61)	4.10 (1.00)
Length	2.90 (1.27)	2.73 (1.22)	3.19 (1.30)	2.89 (1.18)	2.67 (1.22)	2.63 (1.25)
Explanation of risks and benefits	4.36 (0.80)	4.50 (0.80)	4.19 (0.94)	4.56 (0.78)	4.48 (0.67)	4.47 (0.82)
Explanation of consent process	4.49 (0.82)	4.63 (0.76)	4.58 (0.64)	4.89 (0.47)	4.42 (0.94)	4.47 (0.86)
Explanation of right to withdraw	4.63 (0.55)	4.54 (0.77)	4.73 (0.45)	4.67 (0.82)	4.55 (0.62)	4.47 (0.82)
Explanation of available support	3.80 (1.24)	3.65 (1.28)	3.88 (1.21)	3.83 (1.10)	3.73 (1.28)	3.53 (1.38)
Suitability of language	4.22 (1.27)	4.35 (1.12)	4.19 (1.30)	4.33 (1.24)	4.24 (1.28)	4.37 (1.07)
Explanation of data protection^a^	4.53 (0.68)	4.46 (0.74)	4.67 (0.62)	4.72 (0.57)	4.42 (0.71)	4.30 (0.79)
Feasibility of study	4.32 (0.94)	3.85 (1.13)	4.04 (1.15)	4.11 (1.18)	4.55 (0.67)	3.70 (1.09)
Overall composite score	46.69 (5.19)	45.81 (6.10)	46.87 (6.01)	47.89 (5.21)	46.55 (4.54)	44.57 (6.33)

a. Represents a significant main effect of participant group (patients vs controls) on ratings.

#### Does participant group affect ratings?

Regardless of information sheet review status, there was a main effect of participant group type (F(11,93) = 1.98, *P* = 0.039, Wilk's Λ = 0.81, partial η2 = 0.19). This manifested through the description of the protection of participants’ data (F(1,103) = 5.95, *P* = 0.016, partial η2 = 0.06), which the control group rated as significantly worse (M = 4.36 ± 0.09) than patients (M = 4.70 ± 0.11).

#### Does review status affect views between the groups?

Overall, the participant group × pre/post-review interaction approached significance (F(11,93) = 1.81, *P* = 0.063, Wilk's Λ = 0.82, partial η2 = 0.18). This interaction was statistically significant for three items, which the patients rated as improved after review, whereas the control group rated them as worse. The three questions were the ease of language to understand (F(1,103) = 6.71, *P* = 0.011), study feasibility (F(1,103) = 5.23, *P* = 0.024) and structure of the document (F(1,103) = 4.70, *P* = 0.032).

Our sensitivity analysis found no statistical changes, but the main effect of participant type on ratings of protection of participants’ data approached significance (*F*(1,87) = 3.89, *P* = 0.052, partial η2 = 0.04).

#### Qualitative results

**Fig. 1:**
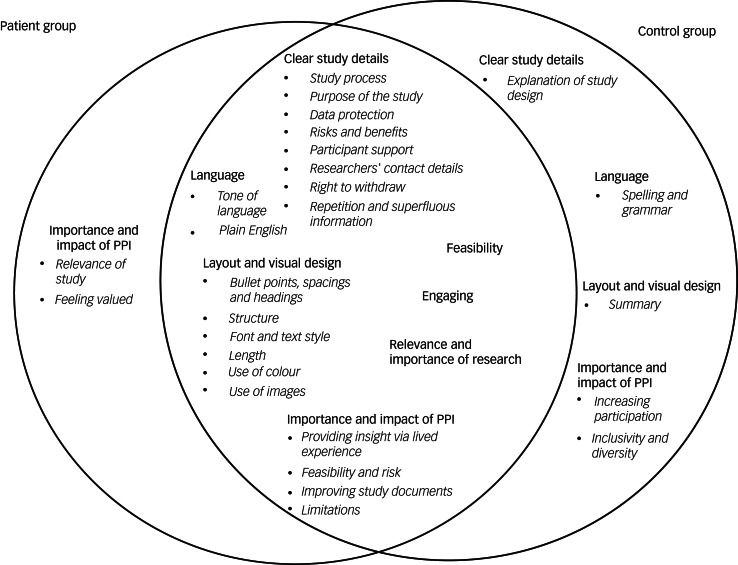
Comparison of emerging themes in the patient group (left) and control group (right).

Thirty-five participants completed the interview (21/21 controls, 14/15 patients). Seven themes with subthemes emerged (Fig. 1). Most themes overlapped between the two groups and mirrored the results of previous studies.^3,8^ However, there were also differences between the two groups, with control group participants focusing more on practical aspects, such as an explanation of the study design, correct spelling and grammar, and having a study summary at the beginning of an information sheet. During the interviews, we explained the concept of PPI to participants, and whereas the control group felt that PPI may lead to increased participation and improve inclusivity and diversity, patients particularly valued how PPI could help those involved feel valued and improve the relevance of the study, ensuring that it brings about meaningful outcomes.

#### Clear study details

Participants valued information sheets that provided clear details on the study process, data protection, the risks and benefits of the study, and how participants are supported if they experience distress during or after the study. Participants particularly highlighted the importance of support for more invasive studies. It was brought up that ‘*MRIs can be quite distressing situations […] what happens if I'm distressed while in the MRI?*’ (Participant 16, control group). The purpose of the study should be highlighted as early as possible in the information sheet, as participants were eager to feel they would be helping achieve research aims. Information sheets should provide researchers’ contact details and clearly outline participants’ right to withdraw from the study. Repetition and superfluous information made the information sheet harder to understand. In addition, control group participants stated that they would like to be informed about the study design (‘*I thought explaining to the participants how the study was designed is quite important, ‘cause it enables the participants […] gives them informed choice to participate in the study*’ Participant 1, control group).

#### Feasibility

Both groups viewed information sheets more favourably if the study itself was feasible and did not ask too much of participants in terms of study duration or invasive tasks (‘*the procedure, I think it's very invasive. Blood tests, faecal matter, nothing I would like to do […] it's a bit complicated when you have to do over two days […] It's a lot for one research. Yes, probably too much*’ Participant 26, patient group).

#### Engaging

Both patients and control participants preferred information sheets that they found engaging and interesting. Highlighting key information as soon as possible was seen as a good way to increase engagement, as well as only providing the key information (‘*I would go for some kind of punchy information right up front that engages the reader and makes it a bit more accessible*’ Participant 27, patient group). An interesting format and colours drew the eye and encouraged participants to read the sheet (‘*I found the colour a lot more engaging*’ Participant 21, patient group).

#### Language

Many participants highlighted issues with the tone of the information sheet, for example, the use of stigmatising, age-inappropriate or ‘*patronising*’ (Participant 10, control group; Participant 40, patient group) language. Information sheets that used a friendly and empathetic tone were viewed more favourably (‘*It was a bit harsh. I feel like I didn't really fit the narrative, and if I did kind of try and see myself in there, I would feel a bit like marginalised*’ Participant 31, patient group). Some language was perceived by participants as ‘*frightening*’ (‘*It said it's shown that even though people are having treatment, they don't get better and because of the way I read things because of my OCD, I know that if I had read that when I was feeling poorly, I'd have just hung on to that and it would have freaked me out*’ Participant 32, patient group). Importantly, information sheets needed to use plain English throughout the document, avoiding jargon as much as possible. Control group participants identified spelling and grammatical errors in some sheets which made them appear less professional.

#### Layout and design

One of the most important aspects influencing the scoring was its layout. Bullet points, headings and spacing helped to break up the text and signpost the reader to the important sections. Font and text style were often mentioned, with participants preferring larger font sizes and suggesting putting headings and important information in bold or italics, to highlight these better. The document length was also said to affect participants’ willingness to engage with it, as overly lengthy and long-winded documents felt daunting to read. The structure of information sheets was key. Participants particularly liked a question-and-answer format with an abstract at the beginning, providing the key information early on (‘*the aim of the project I felt should go first*’ Participant 23, patient group). The use of colour in an information sheet was more aesthetically pleasing; however, it could have the opposite effect when colours were used that made the document harder to read. Images, such as of equipment to be used during the study, including imaging machines, were viewed positively by most participants, as they made the information sheets more enjoyable to read and helped clarify parts of the study process. Including the organisation's logo on the document made the study appear more credible. However, some participants pointed out that overloading the information sheet with images would overwhelm the reader and make the document less visually appealing.

#### Relevance and importance of research

Ratings were higher when the topic was useful, interesting, and novel (‘*It's got to be relevant, it's got to be contemporary […] it's gotta be a new aspect or alternative view*’ Participant 22, patient group). Information sheets should clearly state how the research would be beneficial to society, as participants wanted to know that their participation in the study would be meaningful (‘*I would want to know that there is a valid purpose to it – that it was achieving something*’ Participant 27, patient group). Participants were more interested in taking part if the information sheet described the novelty of the study (‘*One of the things it says: “this has never been done before” or “this will broaden our knowledge” […] I thought OK, that's interesting then because that means I'll be taking part in something that has never been done before, so that I found fascinating*’ Participant 10, control group).

#### Importance and impact of PPI

Most participants did not initially appear to fully understand the meaning or purpose behind PPI and therefore rated it as being less important. Some believed it meant that patients would design and run the study, and there were concerns over the credibility of such studies. When PPI was explained, opinions changed, and it was seen as important by most. Participants felt that when people provide insight into a condition via lived experience, it could improve the overall quality of the study as well as helping to shape its direction. PPI members may prompt research questions otherwise not considered and help speed up the study design process, as well as reducing possible risk to the participants. It was also felt that PPI could improve a study's feasibility and improve the quality of study documents, especially in terms of using non-stigmatising language that is easy to understand.

Control group participants believed PPI could ensure studies were inclusive and diverse and help increase participation, for example, by PPI members referring other participants to the study. Patient participants felt that involving people with lived experience in the study and information sheet design would make people feel that they were valued and their opinions appreciated, as well improving the relevance of the study, by making sure the right questions are being asked and that real-life issues are being investigated. Both groups also pointed out potential limitations with PPI in research. It was suggested that PPI could cause researchers to compromise essential elements of the study and could lead to difficulties in narrowing down the research when presented with too many varying ideas. Some felt the running of the study should be left to researchers, as a layperson may not fully understand the scientific process.

## Discussion

This was a mixed-methods quasi-experimental study investigating opinions of how PPI affects participant information sheets. Patient participants rated information sheets as more accessible after review, although this was not statistically significant, whereas control participants with no mental health service use considered the information sheets to be less accessible. This may be explained by mental health patients having higher mental health literacy than people in the general population, enabling them to understand terms and procedures detailed in the information sheets more easily. However, this should not be relied upon when designing an information sheet, as the sheets should be accessible to everyone. Overall, ratings of information sheets from both before and after review were high, indicating that researchers were abiding by the guidelines set out by regulatory bodies, even before review. This also meant that ratings may have suffered from a ceiling effect, so it was harder to detect differences between before and after review. All measures assessed were rated on average over four out of five, on both occasions, except for support available to participants and length of the document. Researchers should place more importance on clearly describing the support provided, as well as keeping the information sheet as short as possible. We found marginal, non-significant improvements in patient ratings after PPI review. This may have been due to a lack of power, so future work should aim to recruit more patient participants and assess a wider variety of information sheets to increase the power and detect whether significant differences exist.

Despite previous work finding that mental health documents are too complicated for many people to understand,^1,3,20^ overall ratings of the information sheets were high, indicating good levels of accessibility. The only difference between the patient and control groups was ratings of the description of data protection, which patients rated better than controls. Patients have concerns about the protection and sharing of their data,^21,22^ and our findings suggest that researchers are successfully providing the data protection information that patients care most about.

Previous work assessed the impact of PPI review on participant-facing documents^3,8^ by thematically analysing reviewers’ comments on the documents. We found that themes and comments from these studies mirrored the findings of our current study, as well as guidelines set out by regulatory bodies, such as the Health Research Authority. However, these studies did not investigate subjective opinions from before and after review documents. Objective outcomes (e.g. word count, number of jargon words, reading grade) do not consider all aspects of accessibility to participants. Jilka et al^3^ overcame this limitation in a blind preference test of information sheets, but only the views of PPI reviewers were assessed. We have built on these findings by assessing the opinions of research-naïve patients and individuals without a mental health diagnosis.

Both groups rated patient involvement as unimportant. This was corroborated by the qualitative analysis, which found that participants in both groups often misinterpreted the meaning of PPI, with many believing it meant the full running of the study being conducted by non-researchers. When PPI was explained, many changed their minds and described the valuable effects it could have on shaping the research and study documents. This indicates that the true meaning of PPI is not well understood among either the general population or mental health patients – those who are not involved in existing PPI activities. PPI opportunities should be advertised to a larger group of people, rather than a selective group undertaking the majority of PPI work. Further work is needed to increase the public's awareness and understanding of PPI.

Our qualitative data highlight important aspects for researchers to consider in the design of information sheets. Documents should explain the study details as clearly and concisely as possible and use language that is easy to understand. Investing time in making documents as visually pleasing as possible, for example, by breaking up the text into sections, using images and colour to make the document more engaging, reducing the length and using larger fonts, will be valued by potential participants.

### Strengths and limitations

This is the first empirical study to conduct a double-blind experiment assessing the impact of PPI on participant information sheets. Our participant group was ethnically diverse (21/35; 60% from a minority ethnic group). Diversity in PPI is a neglected area,^23^ and we have gained the opinions of a wider group of participants than is common in PPI research,^6,14^ improving the generalisability of our findings. However, as this study employed a relatively small sample size, further research is required.

As the included information sheets came from studies in different study types and fields (e.g. clinical trials, cross-sectional investigations and qualitative studies),^3^ some findings may be artefacts of the information sheets themselves rather than their status as either before or after review. To minimise this, we ensured an even distribution of information sheet complexity and topic area between the two participant groups. There also were differences in the number of changes between the two versions of the same information sheet. Two documents had only three changes after PPI review, which may not be sufficient for participants to identify differences between the two versions. However, this was investigated via sensitivity analyses and was found to not affect the results.

The NIHR emphasises the importance of evaluating the impact of PPI.^24^ We stress that caution must be applied when doing so, as quantitative or qualitative analyses may not tell the whole story when it comes to the value that PPI has in research. We have highlighted that many patients are unaware of PPI, and therefore work must be done to broaden the group of people who contribute to research.

## Data Availability

The data that support the findings of this study are available from the corresponding author, S.J., upon reasonable request.

## Supplementary material

For supplementary material accompanying this paper visit http://doi.org/10.1192/bjo.2022.627. click here to view supplementary material
