# Supplementary material for: The impact of patient involvement on participant opinions of information sheets
Source: BJPsych Open. 2023 Jan 9;9(1):e10. doi: 10.1192/bjo.2022.627 (PMC9885327; doi:10.1192/bjo.2022.627)
Supplement: Supplementary file 1 [file S2056472422006275sup001.docx]

## Methods

### Interview Topic Guide

- Explain that I will start audio recording
- Confirm again when audio recording
- Go over scoring sheets and why rated the information sheet like this

“What made you rate the PIS overall highly/low?” (*Go over individual questions that stand out*)

- Go over study feature importance questionnaire ranking

What affected your decisions when you rated the documents?

Were there any questions that were difficult to rate?

Were there any questions that you did not think were relevant?

Were there any questions which you think are relevant, but we did not consider?

- Now I would like to ask some questions about PPI in relation to research documents (*explain PPI here if needed*)

Do you think PPI is important at the research design stage?

Could you tell if any of the information sheets had had PPI?

What impact do you think PPI has on the research process?

How else do you think we could measure the impact of PPI on patient facing documents?

## Results

### Breakdown of differences in demographic characteristics, by participant group

1. Age - F(1,33) = 1.80, p = .189
2. Highest Qualification - χ2 (4) = 5.71, p = .222.
3. Gender - χ2 (1) = 1.71, p = .191.
4. Ethnicity - White vs POC. χ2 (1) = 0.97, p = .324
5. Employment Status - Employed full time vs not. χ2 (1) = 2.76, p = .096.
6. Native English Speaker - χ2 (1) = 1.64, p = .200.
7. Student Status - χ2 (1) = 1.08, p = .298

#### Service users

| **Supplementary Table 1.** Various aspects of study design and documents that were highlighted by service users as being important. | | |
| --- | --- | --- |
| **Important aspects** | | **References** |
| **Clear study details** | |  |
|  | Study process | 46 |
|  | Purpose of the study | 18 |
|  | Data protection | 16 |
|  | Risks and benefits | 7 |
|  | Participant support | 7 |
|  | Researchers’ contact details | 9 |
|  | Right to withdraw | 4 |
|  | Repetition and superfluous information | 5 |
| **Feasibility** | | 12 |
| **Engaging** | | 12 |
| **Language** | |  |
|  | Plain English | 41 |
|  | Tone of language | 7 |
| **Layout and Design** | | 13 |
|  | Length | 24 |
|  | Bullet points, spacing and headings | 23 |
|  | Structure | 22 |
|  | Use of images | 17 |
|  | Font and text style | 12 |
|  | Use of colour | 8 |
| **Importance and Impact of PPI** | |  |
|  | Providing insight via lived experience | 8 |
|  | Feasibility and Risk | 7 |
|  | Relevance of study | 4 |
|  | Feeling valued | 4 |
|  | Improving study documents & Language | 4 |
|  | Limitations | 15 |
| **Relevance and importance of research** | | 34 |

#### Control Group

| **Supplementary Table 2.** Various aspects of study design and documents that were highlighted by control group participants as being important. | | |
| --- | --- | --- |
| **Important aspects** | | **References** |
| **Clear study details** | |  |
|  | Study process | 107 |
|  | Repetition and superfluous information | 23 |
|  | Purpose of the study | 23 |
|  | Participant support | 16 |
|  | Explanation of study design | 15 |
|  | Data protection | 12 |
|  | Right to withdraw | 10 |
|  | Researchers’ contact details | 7 |
| **Feasibility** | | 15 |
| **Engaging** | | 6 |
| **Language** | |  |
|  | Plain English | 50 |
|  | Tone of language | 15 |
|  | Spelling and grammar | 6 |
| **Layout and design** | | 35 |
|  | Length | 83 |
|  | Bullet points, spacings and headings | 68 |
|  | Font and text style | 27 |
|  | Use of images | 29 |
|  | Structure | 23 |
|  | Use of colour | 13 |
|  | Summary | 6 |
| **Importance and Impact of PPI** | |  |
|  | Providing insight via lived experience | 23 |
|  | Improving information sheets & Language | 8 |
|  | Increasing participation | 6 |
|  | Inclusivity and Diversity | 4 |
|  | Feasibility & Risk | 2 |
|  | Limitations | 16 |
| **Relevance and importance of research** | | 5 |
